# Topological Voting Method for Image Segmentation

**DOI:** 10.3390/jimaging8020016

**Published:** 2022-01-18

**Authors:** Nga T. T. Nguyen, Phuong B. Le

**Affiliations:** 1 Torus Actions SAS, 31400 Toulouse, France; nttnga@torus-actions.fr; 2 Department of Mathematics, Hanoi University of Mining and Geology, Hanoi 100000, Vietnam

**Keywords:** voting method, image segmentation

## Abstract

Image segmentation is one of the main problems in image processing. In order to improve the accuracy of segmentation, one often creates a number of masks (annotations) for a same image and then uses some voting methods on these masks to obtain a more accurate mask. In this paper, we propose a voting method whose voting rule is not pixel-wise but takes into account the natural geometric-topological properties of the masks. On three concrete examples, we show that our voting method outperforms the usual arithmetical voting method.

## 1. Introduction

Voting methods are ubiquitously used in human and artificial intelligence to improve the accuracy of automatic as well as hand-made annotations. The theoretical reason is simple: assuming that most individual annotators are relatively good (more often right than wrong) and independent to some extent, then for most situations, a majority of the annotators will be right while only a minority will be wrong, so by voting we are more likely to obtain a correct annotation in more situations than most individual annotators. Of course, when there are too many bad annotators who do not know what they are doing, then voting methods may be counter-productive: the results of good annotators will be killed by the results of bad annotators.

In this paper, we present our research on a novel voting method for the image segmentation problems in AI suggested to us by Professor Nguyen Tien Zung, Founder of Torus Actions SAS. This problem has been extensively explored in many papers [1,2,3,4,5,6,7,8] and the recently in the article [9]. The most popular voting method for image segmentation is the (soft or hard) arithmetical voting [4,5,6], where each pixel is voted by a majority rule for that pixel only and does not take into account the other pixels. Our starting idea is that the masks in each natural segmentation problem have a natural geometric-topological structure, the pixels are interrelated and not independent, so each pixel should be voted not individually but in connection with the other pixels. That is why we call our method the *topological voting method*. The idea of considering the structure of the mask rather than pixel-wise using in ensemble method has been studied in [1,2,3], where an image is divided into several regions (clusters) and the voting methods are applied for the regions. We shall discuss more about this idea in Section 2 of this paper.

We will present the topological voting algorithm, together with its variations, in Section 2. We will show not just one, but a whole family of topological voting methods, including *local* topological voting, and *hybrid* voting, which is a combination of arithmetical and topological voting methods, with a step to detect and exclude the outliers, those who are more likely to be wrong.

For simplicity, in this paper we will consider only the binary segmentation of 2D images, i.e., each image will be segmented into two parts: the region of interest, called the *mask*, and the rest (the background). To illustrate our ideas, we will work out three concrete examples: (1) segmentation of salt in seismic images; (2) segmentation of human faces in photos; and (3) segmentation of blood vessels in retinal images. These three examples are pretty typical of segmentation problems, and in all these examples, the experimental results, presented in Section 3, Section 4 and Section 5 of this paper, show that the topological voting method and its variations allow one to achieve better accuracy than the arithmetical voting method.

In Section 6, we offer some arguments, theoretical and experimental results that show *why* topological voting methods are efficient and give better results than arithmetical ones in many situations. These are the arguments and ideas that led to our experiments.

Section 7, the last section of this paper, is dedicated to conclusions and future work.

## 2. The Topological Voting Method

### 2.1. Image Segmentation and Jaccard Distance

Mathematically, one may represent a 2D-image binary segmentation tool or agent (a segmentor) as a discrete-valued map

(1) $$S:\Omega \to {\left\{0,1\right\}}^{h\times w}$$

or a continuous-valued map

(2) $$\tilde{S}:\Omega \to {\left[0,1\right]}^{h\times w}$$

from a space Ω of digital 2D images (of a fixed size, for simplicity), and N=h×w (height times width) is the number of pixels per image. For an image x∈Ω, if S(x)(i,j)=1, where (i,j) is the position of a pixel (1≤i≤h,1≤j≤w), then it means that this pixel is in the mask of *x* made by the segmentor *S*; otherwise, the pixel is the background.

Convolutional neural networks (CNNs) that are used in image segmentation problems usually give us continuous-valued segmentors $\tilde{S}$, and we can obtain *S* from $\tilde{S}$ by fixing a threshold, for example

(3) $$S\left(x\right)\left(i,j\right)=\left\{\begin{array}{c} 0\;\mathrm{if}\;\tilde{S}\left(x\right)\left(i,j\right)<0.5\\ 1\;\mathrm{if}\;\tilde{S}\left(x\right)\left(i,j\right)\geq 0.5 \end{array}\right.$$

We will assume that each image x∈Ω has a true mask (the ground truth) denoted by $S_{\mathrm{true}}\left(x\right)$. We want to measure how good our segmentor is, i.e., how far is the mask S(x) from the true mask $S_{\mathrm{true}}\left(x\right)$.

A good and widely used measure of accuracy of binary segmentation is the so-called *intersection over union* (IOU) score, also known as the *Jaccard score*, introduced by Paul Jaccard in a paper in 1901 [10].

(4) $$J\left(S\left(x\right),S_{\mathrm{true}}\left(x\right)\right)=\frac{|S\left(x\right)\cap S_{\mathrm{true}}\left(x\right)|}{|S\left(x\right)\cup S_{\mathrm{true}}\left(x\right)|}$$

where $S\left(x\right)\cap S_{\mathrm{true}}\left(x\right)$ denotes the intersection of the two masks S(x) and $S_{\mathrm{true}}\left(x\right)$ (i.e., the set of pixels where both segmentors have value equal to 1), $S\left(x\right)\cup S_{\mathrm{true}}\left(x\right)$ denotes their union (where at least one of them has value equal to 1), and the absolute value sign denotes the surface area (i.e., the number of pixels in the set).

Remark that an equivalent way to write the Jaccard score is MOM (min over max):

(5) $$J\left(S\left(x\right),S_{\mathrm{true}}\left(x\right)\right)=\frac{\sum_{i,j}\min\left(S\left(x\right)\left(i,j\right),S_{\mathrm{true}}\left(x\right)\left(i,j\right)\right)}{\sum_{i,j}\max\left(S\left(x\right)\left(i,j\right),S_{\mathrm{true}}\left(x\right)\left(i,j\right)\right)}$$

An advantage of Formula (5) over Formula (4) is that it also works for *soft masks*, i.e., for continuous-valued segmentors: if *U* and *V* are two soft masks then we can define their relative *soft Jaccard score* as

(6) $$J\left(U,V\right)=\frac{\sum_{i,j}\min\left(U\left(i,j\right),V\left(i,j\right)\right)}{\sum_{i,j}\max\left(U\left(i,j\right),V\left(i,j\right)\right)}$$

The *Jaccard distance* of a mask S(x) to a true mask $S_{true}\left(x\right)$ is defined by the formula

(7) $$d_{Jaccard}\left(S\left(x\right),S_{\mathrm{true}}\left(x\right)\right)=1-J\left(S\left(x\right),S_{\mathrm{true}}\left(x\right)\right),$$

and it measures “how far” S(x) is from $S_{\mathrm{true}}\left(x\right)$. The two masks coincide if, and only if, the Jaccard distance between them is equal to 0. In the case of soft masks, the same formula still works and gives what we call the *soft Jaccard distance*.

In this paper, we will mainly use the Jaccard score (and the Jaccard distance) to measure the accuracy of our automatic segmentors.

Let us mention that there is a naive *binary accuracy score*, which is by definition the number of pixels at which S(x) coincides with $S_{\mathrm{true}}\left(x\right)$ divided by the total number of pixels *N*. When the true mask is small (the problem of segmentation of small objects), it may happen that the segmentor *S* gives a mask which is completely different from the true mask (no intersection) and the binary accuracy score is still near 1 (maximal possible), so this binary accuracy score is not a very good measure of accuracy, though we may still compute it sometimes.

### 2.2. Arithmetical Voting

In automatic as well as hand-made segmentation, one often creates not just one, but many segmentors $S_{1},\cdots ,S_{n}$ for the same problem, using different CNNs, or different training datasets, or different data augmentation methods, etc., and then ensembles them by a voting method to hopefully obtain a segmentor which is more accurate on the average than each one of them. The most obvious voting method is the majority vote: for each pixel, each segmentor has one vote, and the candidate value (0 or 1) that has the most votes wins. This majority voting method is also called the *hard arithmetical voting*, and there is another variant of arithmetical voting called the *soft voting*, see, e.g., [4,5]. In soft voting, one uses continuously-valued segmentors ${\tilde{S}}_{1},\cdots ,{\tilde{S}}_{n}$ instead of discrete-valued segmentors $S_{1},\cdots ,S_{n}$, and put

(8) $${\tilde{S}}_{\mathrm{voted}}=\frac{1}{n}\sum_{k=1}^{n}\tilde{S_{k}}$$

when *n* is large, then by the law of large numbers, soft voting and hard voting will give more or less the same results. However, when *n* is small, soft voting may be finer and give slightly better results than hard voting. One may fine-tune the above arithmetical voting formula by giving different weights to different segmentors (weighted averaging formula).

Our *topological voting methods* are very different from arithmetical voting. In the following Section 2.3, Section 2.4 and Section 2.5 we present three versions of the method. One of them—the simplest version of the proposed method, turns out to be the same as the “Best of K” method in [2], though we arrived at it independently. The other two versions are different.

### 2.3. Topological Voting: Simplest Version

The simplest forms (hard and soft topological voting) are presented in the following. The hard version is the one called “Best of K” in [2] with only one cluster that is the whole image. More details, they both consist of the following steps:

(i) For an input image *x*, take *n* masks $S_{1}\left(x\right),\cdots ,S_{n}\left(x\right)$ given by *n* different segmentors $S_{1},\cdots ,S_{n}$;
(ii) For each index k∈{1,⋯,n}, measure the total distance from $S_{k}\left(x\right)$ to the other masks, with respect to some natural distance function.
  There are different natural distances in geometry that fit different problems. For example, the Hausdorff distance, also known as Hausdorff–Pompeiu distance, can be used effectively in many image processing problems [11,12]. For simplicity, here, we will only use the (soft or hard) Jaccard distance in our experiments and define the total distance $d_{k}\left(x\right)$ from $S_{k}\left(x\right)$ to the other masks by the following formula:
  (9) $$d_{k}\left(x\right)=\sum_{i=1}^{n}d_{Jaccard}\left(S_{i}\left(x\right),S_{k}\left(x\right)\right)$$
(iii) (Winner takes all) The mask with the smallest total Jaccard distance to the other masks wins, i.e.,
  (10) $$S_{\mathrm{voted}}\left(x\right)=S_{l}\left(x\right)\;\mathrm{where}\;l=\underset{k}{arg min}d_{k}\left(x\right)$$
  An equivalent way to formulate the “winner takes all” rule is: the mask with the highest total Jaccard score wins, i.e.,
  (11) $$S_{\mathrm{voted}}\left(x\right)=S_{l}\left(x\right)\;\mathrm{where}\;l=\underset{k}{arg max}J_{k}\left(x\right)$$
  and
  (12) $$J_{k}\left(x\right)=\sum_{i=1}^{n}J\left(S_{i}\left(x\right),S_{k}\left(x\right)\right)$$

In *soft topological voting*, one uses the same formulas as above but applied to the soft masks instead of the hard masks. A technical side notes: it may be useful to regularize the sigmoid values of the soft masks before computing soft scores, for example, by truncating them at 0.2 and 0.8: $s_{regularized}=0$ if 0≤s≤0.2, $s_{regularized}=10\left(s-0.2\right)/6$ if 0.2≤s≤0.8, and $s_{regularized}=1$ if 0.8≤s≤1. This is actually what we do with soft masks in our experiments.

It turns out that, in many cases, the above “winner takes all” simple topological voting method already gives results that are superior to the arithmetical voting method. We will show it in the example of salt segmentation in seismic images (see Section 3).

The following is a diagram for the system:
Topological Voting Schema (Simplest Version)  Candidates $Y_{1},Y_{2},Y_{3},...,Y_{n}$ (Topological objects)  Topological distances $d_{ij}=d\left(Y_{i},Y_{j}\right)$  Total distance from $Y_{i}$ to the rest$$d_{i}=\Sigma_{j}d_{ij}$$Selected candidate *Y selected*, where $\;\mathrm{selected}={arg min}_{i}d_{i}$


### 2.4. Local Topological Voting

Some objects (e.g., blood vessels) have a complicated global structure and so even a good segmentation may be imprecise in many places. To improve the accuracy of annotation, one should in theory reduce the complexity of the things to annotate by decomposing them into smaller, simpler things. For image segmentation, it means that we can often cut a big complicated image into smaller, simpler ones, easier to segment. This ‘localized segmentation’ strategy together with the topological voting idea leads to what we call *local topological voting*.

To be more concrete, the local topological voting algorithm that we will use for our experiments presented in this paper is the following:

(i) Fix a natural number *s*, which will be the *radius* of the local neighborhoods;
(ii) For each pixel, consider the neighborhood of radius *s* around that pixel: If the pixel is at position (x,y), then its *s*-neighborhood will be the square [x−s,x+s]×[y−s,y+s]. (If the pixel is near the border, then its *s*-neighborhood will be the intersection of this square with the image);
(iii) Use the topological voting algorithm, as presented in the previous subsection, on the predicted masks restricted to the *s*-neighborhood of the pixel (x,y) to obtain the result for this pixel. In other words, the voted value of the pixel (x,y) is equal to the value at (x,y) of the annotator which is considered to be locally topologically the best in the *s*-neighborhood of (x,y);
(iv) Do the above step (iii) for every pixel to obtain the total mask.

The above local topological algorithm contains a parameter *s*, which is the size of the neighborhoods, i.e., the degree of locality. Notice that, when s=0, then the *s*-neighborhood of a pixel is just itself, no topological structure is taken into account, and the 0-neighborhood topological voting is just the usual arithmetical voting. At the other extreme, when *s* is greater than the size of the image, then any *s*-neighborhood is the whole image, and we return back to the first version of our topological voting method. By varying *s* from 0 to infinity, we obtain a whole family of voting methods that goes from arithmetical (pixel-by-pixel) to topological (whole-picture) voting.

Intuitively, for each problem there is an optimal neighborhood size, and the two extreme sizes (0 and infinity) are not the best ones. In our experiments, we will vary the radius *s*, and not surprisingly, we will see that some neighborhood sizes are really better than the others (see Section 5 on blood vessel segmentation).

**Figure 1:**
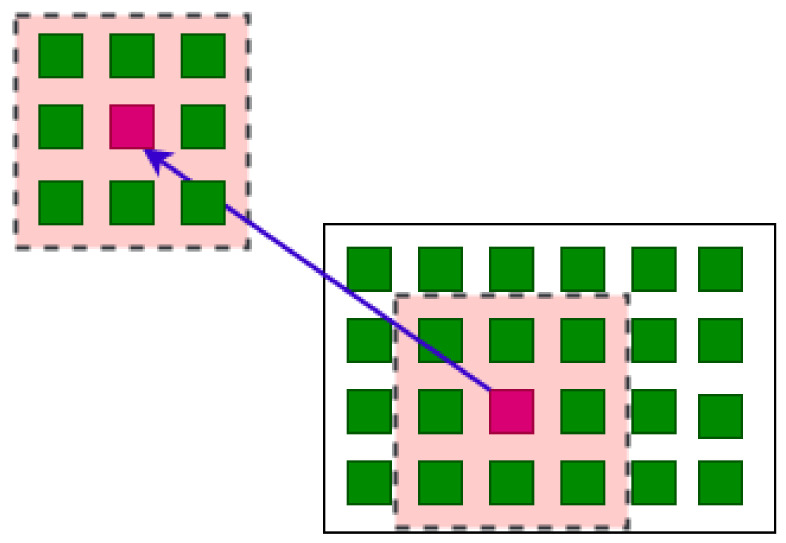
An example for local topological voting s=1.

We illustrate an example of this voting version in Figure 1. In that example, the radius is fixed s=1. At each pixel, a window of that radius is applied for all the segmentators $S1,S_{2},...,S_{n}$ to obtain *n* windows. We then use the method described in Section 2.3 for the *n* windows to obtain the final window. The value of the center pixel of the final window is chosen as the result for the global mask. We repeat these steps through all pixels of the image to obtain the final mask.

### 2.5. Hybrid Topological-Arithmetical Voting

**Figure 2:**
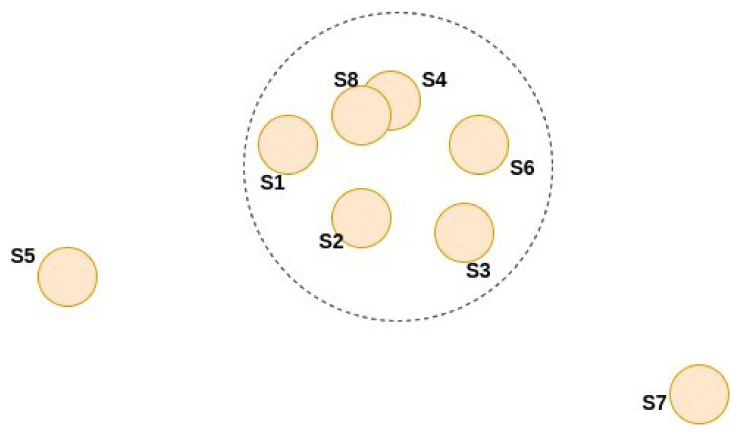
An example for hybrid voting. At round-1, segmentator 5 and segmentator 7 will be excluded since they are far from the others, the rest are kept for round-2 using (either soft or hard) arithmetical voting.

The hybrid voting that we present in this paper is a 2-round voting. An example is illustrated in Figure 2 and it goes as follows:

(i) In the first round, we use the (local or global) topological voting, not to choose the winner, but to choose the losers to exclude from the race.

More concretely, in this paper we will use one of the two following simple exclusion rules for each hybrid voting:

- Method 1: Choose a threshold H>1. For each input *x* keep the masks $S_{k}\left(x\right)$ such that $J_{k}\left(x\right)\geq n/H$, where $J_{k}\left(x\right)=\sum_{i=1}^{n}J\left(S_{k}\left(x\right),S_{i}\left(x\right)\right)$ and *n* is the total number of our individual segmentors. If $J_{k}\left(x\right)<n/H$ then $S_{k}\left(x\right)$ considered to be an “outlier” and is excluded from the second round;
- Method 2: Choose a number $1\leq n_{\mathrm{select}}\leq n$. For each input *x* keep the $n_{\mathrm{select}}$ masks $S_{k}\left(x\right)$ with the highest Jaccard scores $J_{k}\left(x\right)$, and exclude the other $n-n_{\mathrm{select}}$ masks.

In method 1, the number of masks admitted to the second round is not fixed and it is different for each input, while in method 2, this number is always equal to $n_{\mathrm{select}}$.

(ii) The candidates that remain in the second round will be voted arithmetically.

Another variation of hybrid voting is voting with weights determined by the distance function, or equivalently, by the Jaccard scores $J_{1}\left(x\right),\cdots ,J_{n}\left(x\right)$: the higher the Jaccard score $J_{k}\left(x\right)$ is (relatively to the Jaccard scores of the other annotators), the higher the weight of $S_{k}\left(x\right)$ will be in the weighted arithmetical voting formula. Topological voting is when all the weights are equal to 0 except one weight which is equal to 1.

## 3. Salt Segmentation in Seismic Images

The automatic segmentation of salt deposits in seismic images is an important problem for geology companies in search of hydrocarbons. In 2018, Kaggle organized the “TGS Salt Identification Challenge” on this problem, and provided a training dataset of 4000 annotated grayscale images of size 101×101 [13].

Our aim here is not to build a “state-of-the-art” automatic segmentor for the Kaggle challenge (in real life, one will not cut the seismic images into very small pieces and compute the accuracy scores the way Kaggle did anyway), but just to do experiments on the topological voting method. For that purpose, we will build our AI models based on a popular light-weight convolutional neural network architecture called MobileNet [14], which is very handy in the sense that one can train it very fast, and it can run on small devices, such as mobile phones. We train our models using Tensorflow and Keras [15]. The loss function that we use in training our model is the sum of the binary cross entropy and the Dice loss function [16]. We use random padding, translation, rotating, flipping, and cutting, to create square images of size 128×128 from the original images of size 101×101, since our MobileNet models use inputs of size 128×128.

We divide 4000 annotated images into 2 sets: the training set (3000 images) and the test set (1000 images). The results that we show in the tables below are for the test set (not used in the training process of course). The training set is then divided into 5 folds, each fold containing 600 images. For each fold, we train a corresponding AI model, which uses that fold for validation and the other four folds for training. We train each model 500 epochs, each epoch has 3000 inputs, so in total each model is trained on 1,500,000 inputs. Each input is an image taken randomly from the training set and then undergoing random transformations (augmentations in data pre-processing). We do not take the AI model after exact 500 epochs of training, but rather the AI model after the epoch that offers the highest Jaccard scores on validation among all the 500 epochs.

After the above training process, we obtain 5 AI segmentors, corresponding to our 5 folds. Then, for each image in the test set, we vote on the 5 masks given by these 5 segmentors, using arithmetical voting and the (soft and hard) topological voting methods. The results are shown in Table 1. Here, the Jaccard score is the means Jaccard score over the test set.

**Figure 3:**
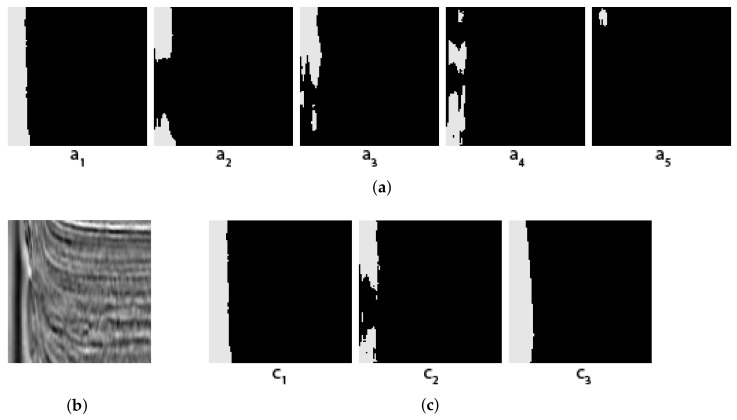
Topological vs. Arithmetical voting in salt segmentation—The first example. (**a**) (Masks created by 5 individual segmentors (from 5 folds); (**b**) Original image; (**c**) $C_{1}$: Topological voting, $C_{2}$: Arithmetical voting, $C_{3}$: True mask.

**Figure 4:**
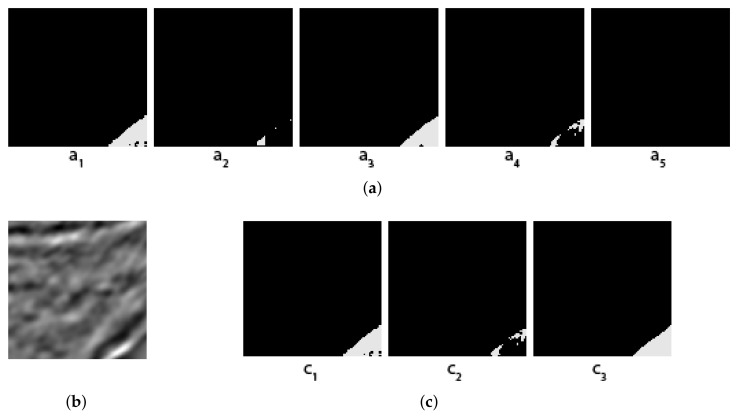
Topological vs. Arithmetical voting in salt segmentation -The second Example. (**a**) Masks created by 5 individual segmentors (from 5 folds); (**b**) Original image; (**c**) $C_{1}:$ Topological voting, $C_{2}:$ Arithmetical voting, $C_{3}:$ True mask.

**Figure 5:**
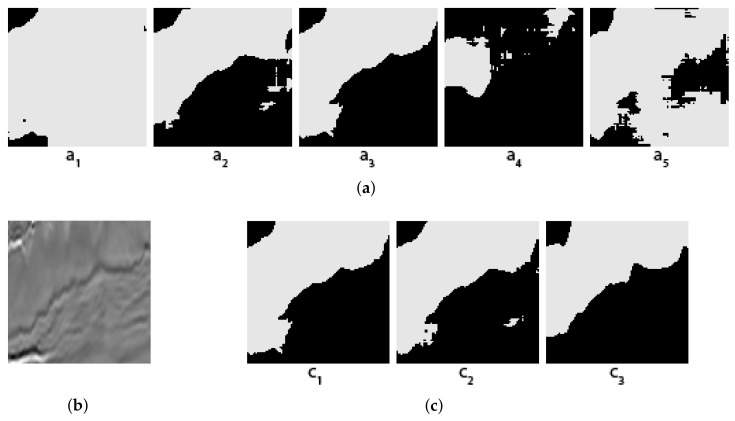
Topological vs. Arithmetical voting in salt segmentation - The third example. (**a**) Masks created by 5 individual segmentors (predictions of the 5 folds); (**b**) Original image; (**c**) $C_{1}$: Topological voting, $C_{2}$: Arithmetical voting, $C_{3}$: True mask.

**Figure 6:**
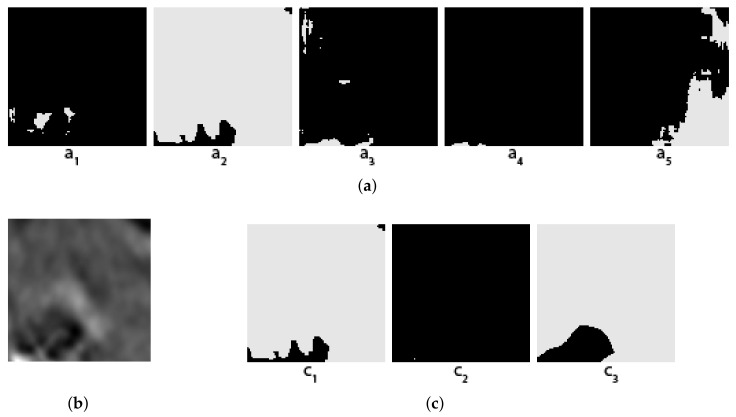
Topological vs. Arithmetical voting in salt segmentation—The fourth example. (**a**) Masks created by 5 individual segmentors (from 5 folds); (**b**) Original image; (**c**) $C_{1}$: Topological voting, $C_{2}$: Arithmetical voting, $C_{3}$: True mask.

Some concrete examples of the masks given by our five individual segmentors, and the results of two different voting methods (arithmetical and topological) and shown in Figure 3, Figure 4, Figure 5 and Figure 6, together with the original images and the true masks (the ground truth given in the dataset). These four figures illustrate how the topological voting method works differently from the arithmetical one. We can see in these figures that the arithmetical chooses the one that equals to average of all mask pixel-wise regardless the structure of each individual mask, consequently, it causes a final voting result which is unstructured regarding that the region of salt is often smooth and continuous. Meanwhile, the topological chooses the mask which is the most common to the others in the meaning that it has the smallest “distance” to the other masks and it is thus reasonable close to the true mask.

In order to improve the accuracy, one can increase the number of individual segmentors. So we created 5 additional AI models using 5 additional folds, in the same way as before. The ensemble results using 10 models, shown in Table 2, are indeed better than the ensemble results using just 5 models.

Table 2 also shows that, for the salt segmentation problem, the topological voting method clearly beats the arithmetical voting method (by more than a full percentage point). This table also shows the results of the hybrid topological-arithmetical voting method (at different thresholds), which are slightly better than the simple topological voting method.

As a side remark, we note that, if we measure the performance by using the binary accuracy metric instead of the Jaccard score, then the scores will be very high even for completely wrong segmentations, and the topological voting method will give worse results than the arithmetical voting method if we use this binary accuracy metric, see Table 3.

## 4. Human Face Segmentation in Photos

The Face and Skin Detection Database which contains 4000 images is created by S. L. Phung, T. Y. Ke, and F. H. C. Tivive and used in [17] to support research on skin segmentation and face recognition. The dataset provides many types of ground-truth, such as human face segmentation and skin segmentation. Here, we will use the human face segmentation of this dataset.

**Figure 7:**
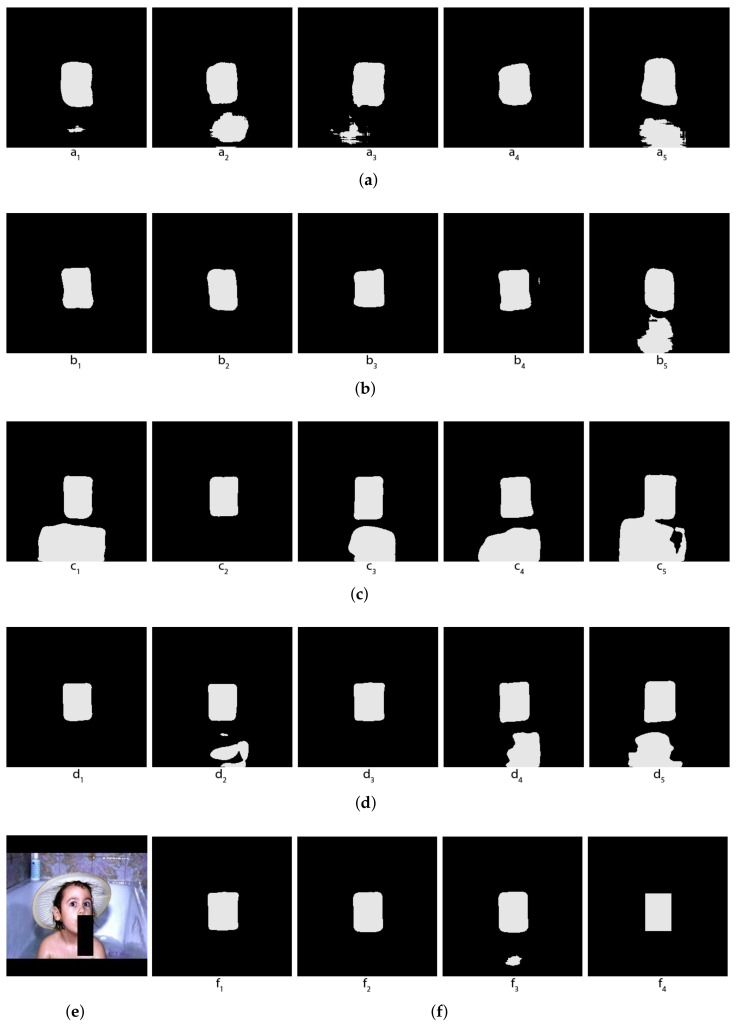
Comparison of voting methods for human faces—The first example. (**a**) Predictions of the first 5 folds (with Mobilenet); (**b**) Predictions of the last 5 folds (with Mobilenet); (**c**) Predictions of the first 5 folds (with EfficienNet); (**d**) Predictions of the last 5 folds (with EfficienNet); (**e**) Original image; (**f**) $f_{1}$: Topological voting, $f_{2}$: Hybrid voting (threshold = 2), $f_{3}$: Arithmetical voting, $f_{4}$: True Mask.

Actually, the masks that we will use are not exactly the human faces, but the smallest rectangles containing them. To make the problem more interesting, we will partially hide the human faces on the photos by random rectangles, and let the machine learn to segment the full human faces despite those hidden parts. Figure 7e shows the original image in which the faces are covered by a random rectangle and Figure 7f (the rightmost image) shows the mask of that image.

The dataset of 4000 photos is divided into 2 subsets: 1000 images for testing, and 3000 images for training. The 3000 training images are divided into 10 folds, each fold contains 600 images, so the folds overlap: the first 5 folds are a partition of the training data, and the second 5 folds are another partition as well.

The original photos in the dataset are of different sizes, but we will augment and resize them into images of size 256×256 before feeding them into our CNN models. The transformations that we use to augment the images are the standard ones: random rotation, flipping, brightness modification, cropping, resizing, padding, and noise adding. As mentioned above, we also add a random back rectangle to each image to partially cover the faces (without changing the masks).

We use two different CNN architectures for our experiment: the light-weight MobileNet [14], and the more heavy-weight EfficientNet B4 [18], so in total, we obtain 20 different AI segmentors for our human face segmentation problem.

It is interesting to look at various inputs and outputs to see how the individual segmentors perform, and what are their main structural mistakes. For example:

In Figure 7, one can see that some individual segmentors mistake body skin for facial skin, while the other segmentors do not make this mistake. Topological and hybrid voting allows us to exclude those segmentors who make this mistake, and so the voted result does not contain body skin in the mask, while the arithmetical voting does not have this semantic advantage, and the result of the arithmetical voting still contains body skin.

**Figure 8:**
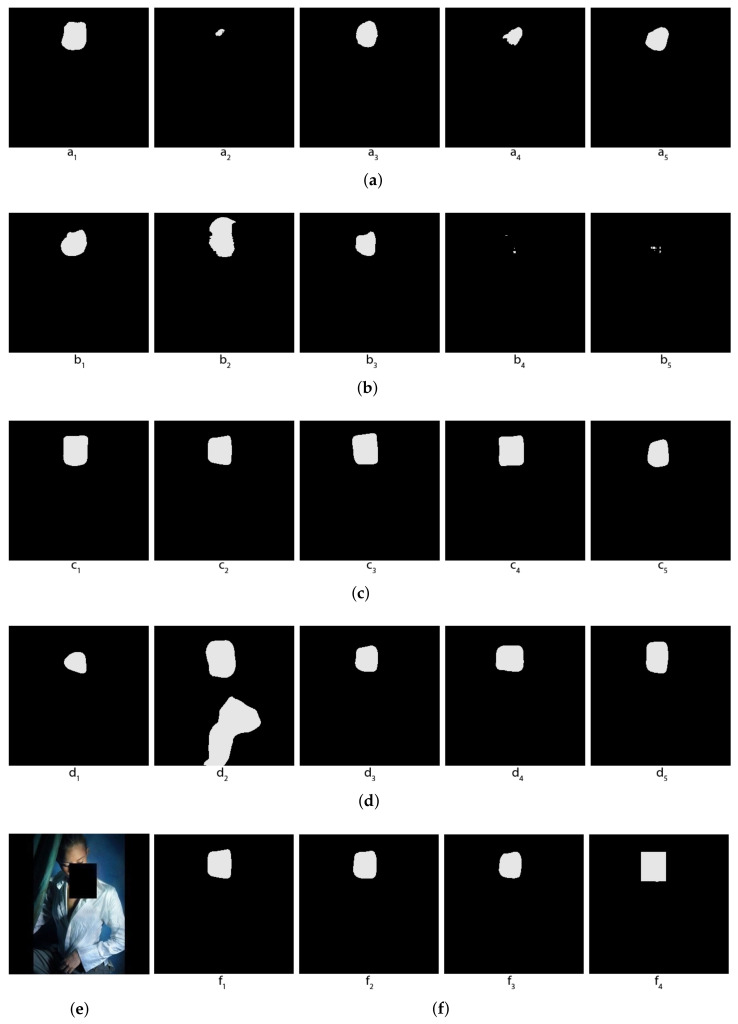
Comparison of voting methods for human faces—The second example. (**a**) Predictions of the first 5 folds (with Mobilenet); (**b**) Predictions of the last 5 folds (with Mobilenet); (**c**) Predictions of the first 5 folds (with EfficienNet); (**d**) Predictions of the last 5 folds (with EfficienNet); (**e**) Original image; (**f**) $f_{1}$: Topological voting, $f_{2}$: Hybrid voting (threshold = 2), $f_{3}$: Arithmetical voting, $f_{4}$: True Mask.

**Figure 9:**
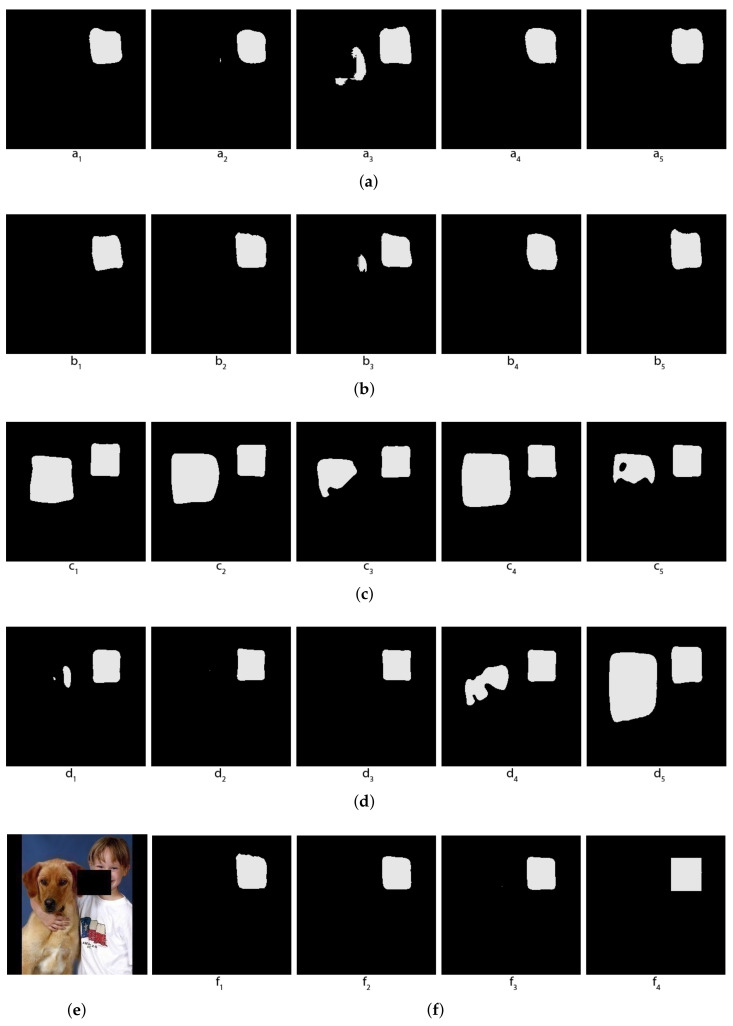
Comparison of voting methods for human faces—The third example. (**a**) Predictions of the first 5 folds (with Mobilenet); (**b**) Predictions of the last 5 folds (with Mobilenet); (**c**) Predictions of the first 5 folds (with EfficienNet); (**d**) Predictions of the last 5 folds (with EfficienNet); (**e**) Original image; (**f**) $f_{1}$: Topological voting, $f_{2}$: Hybrid voting (threshold = 2), $f_{3}$: Arithmetical voting, $f_{4}$: True Mask.

In Figure 8, one can see that some individual segmentors barely recognize any facial skin, while there is one segmentor that mistakes a shirt for facial skin. Figure 9 is another very interesting example, where some segmentors mistake a dog face for a human face.

The accuracy scores of different voting methods on our 20 models are given in Table 4. It shows that hybrid voting gives the best results, by excluding those segmentors that make very gross mistakes.

One may wonder why the plain-vanilla topological voting method gives lower scores than the arithmetical voting method for the above experiment of facial segmentation? We think that the reason lies in the fact that the masks themselves are very simple (just rectangles), and the topological voting method excels at more complicated masks.

## 5. Blood Vessel Segmentation

The problem of segmentation of tree-like structures, such as microglia extensions, neurovascular structures, blood vessels, and pulmonary trees, are of great interest in medical AI, see, e.g., [19] and references therein. Rouchdy and Cohen [19] studied the problem of segmentation of blood vessels in retinal images using a method called *geodesic voting with radius* (no deep learning), and showed the superiority of their method to older approaches such as the edge-based level set method [20], the Chan and Vese method [21], and the fuzzy connectedness method [22]. The database that they used is the digital retinal images for vessel extraction (DRIVE) data [23].

In this section, we propose to use deep learning to solve the retinal blood vessel segmentation problem using this same DRIVE dataset. Again, we will use the light-weight MobileNet [14]. Not surprisingly, the deep neural networks can give better segmentation results than the previous image processing methods, including the geodesic voting method [19].

The DRIVE dataset contains 20 images of size 565×584. We first divide it into two sets: 15 images for training and 5 images for testing. We create 15 folds for the training and the cross-validation of our AI using the 15 training images. Each fold leads to one segmentor, so in total we have 15 individual segmentors to vote on. Each fold uses 13 images for training and 2 images for validation. Each image is then transformed (augmented) using a combination of operations, including random rotating, flipping, padding, brightness modification, noise adding, cropping, and padding, into square images of size 256×256, before being fed into our CNN based on MobileNet for training and validation. The five original images used for testing are cropped into 2000 images of size x×y where *x* and *y* are random whole numbers in the interval [220,256] and then padded to square images of size 256×256, so our test set consists of 2000 images of size 256×256.

**Figure 10:**
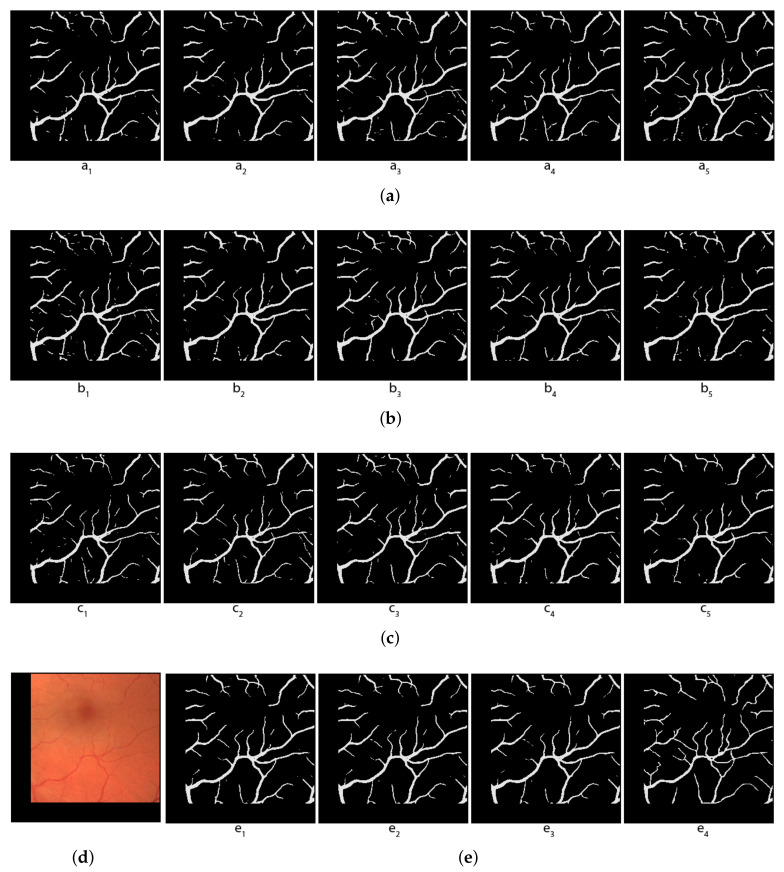
Comparison of voting methods for blood vessel segmentation. (**a**) Predictions of the first 5 folds; (**b**) Predictions of the second 5 folds; (**c**) Predictions of the last 5 folds; (**d**) Original image; (**e**) $e_{1}$: Local topological voting with s=10, $e_{2}$: Hybrid voting (s=10, threshold = 2), $e_{3}$: Arithmetical voting, $e_{4}$: True Mask.

Figure 10 shows an example of our 15 individual segmentations of an image from the test set, and the results of three different voting methods on these 15 segmentations compared to the true mask. One can see visually on these pictures that both the local topological method and the hybrid voting methods give better results than the arithmetical voting methods: fewer missing pixels compared to the true mask (the vessels are less broken).

Table 5 shows a comparison of voting methods on the DRIVE dataset.

One may notice that the Jaccard scores obtained in blood vessels segmentation are much lower than in the previous two problems. The main reason is simple: the vessels are very thin, and so the Jaccard scores are very sensitive to small variations in the segmentation. Another reason is that MobileNet is a light-weight CNN aimed at quick processing and not highest accuracy, and we did not do any special optimization here either. What is important for us here is the fact that the gain obtained by the hybrid topological-arithmetical voting method is significant compared to the arithmetical voting method.

## 6. Why Does Topological Voting Work?

Let us recall that the philosophical reason behind our topological and hybrid voting methods is the following: for meaningful objects, their masks must have certain geometrical or topological properties or shapes. Assuming that we have good (but not yet excellent) segmentors $S_{1},\cdots ,S_{n}$, most masks given by them (for a typical input) will have reasonably good shapes reflecting topological-geometrical properties of the segmented object and are sufficiently close to the true mask. Consequently, in general, a mask which is far from the other masks will also be far from the true mask, while a mask which is closer to most other masks will also be closer to the true mask. So, even though we do not know what the true mask is, we can use the total distance function as a proxy to estimate how far is $S_{k}\left(x\right)$ from the true mask.

As a side remark, one may see a parallel between the different voting methods used in image segmentation and the voting methods used in politics. For example, elections of village mayors are similar to pixel-by-pixel voting (each village is a ‘pixel’), while many presidential elections are similar to whole-picture voting.

Considering the simplest form of the proposed method presented in Section 2.3, in small dimension settings, we show below theoretical and experimental results to explain why our voting method outperforms the classical arithmetical voting method. More detail, when $S_{i}$ is a scalar in R for all *i*, the two voting methods behave similarly, we shall prove that they both converge to θ with the same the rate of convergence $\sqrt{n}$. The difference can be seen when $S_{i}$ is a vector and its components are dependent to describe the ’structure’ inside it. We illustrate below the simulations for the cases when $S_{i}$ is vector in $R^{2}$ and its second component totally depends on its first component to show how our voting method behaves better than the arithmetical one.

### 6.1. One Dimension Case

**Figure 11:**
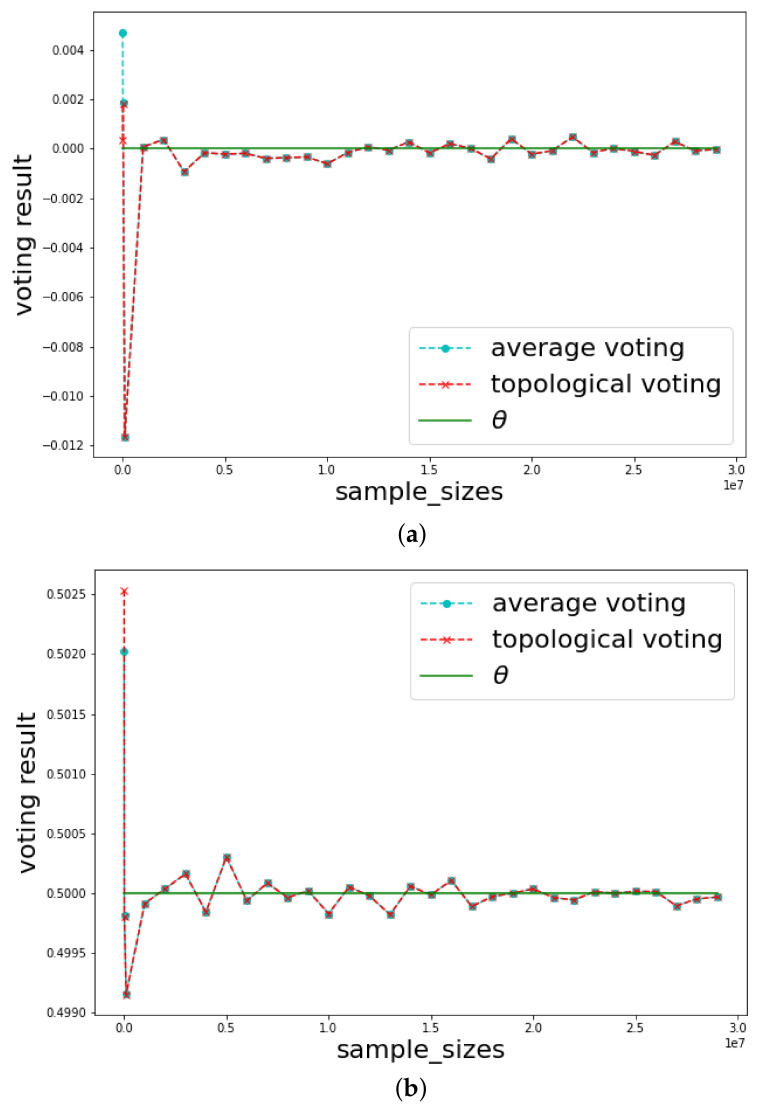
Comparisons of arithmetical voting (average voting) and topological voting method in one dimension setting. (**a**) ${\left\{S_{i}\right\}}_{i=1}^{n}$ are i.i.d. and follow N(0,1); (**b**) ${\left\{S_{i}\right\}}_{i=1}^{n}$ are i.i.d. and follow U(0,1).

In this case, ${\left\{S_{i}\right\}}_{i=1}^{n}$ are i.i.d. variables value in R with mean θ and variance σ. Recall that the classical soft arithmetical voting method (average voting) chooses $\Sigma_{n}=\frac{1}{n}\left(\sum_{i=1}^{n}S_{i}\right)$ as its final result, while our voting policy chooses

$$Y_{n}=\underset{S_{i}\in \left\{S_{1},S_{2},...,S_{n}\right\}}{arg min}\sum_{j=1}^{n}d\left(S_{i},S_{j}\right)$$

where *d* is some distance function. Here, we choose $d\left(x,y\right)={\left(x-y\right)}^{2}$ to obtain a smooth function for analysis. The classical results show that $\Sigma_{n}$ converges almost surely to θ (law of large number) and it converges in distribution to $N(\theta ,\sigma^{2})$ with the rate of $\sqrt{n}$ (central limit theorem). With our voting method, we obtain the same guarantees for the convergence, as well as the rate of convergence, which are illustrated in Figure 11 and showed in Proposition 1. Remark that in Proposition 1, we restrict ourself by assuming that ${\left\{S_{i}\right\}}_{i}$ have positive, continuous, and bounded probability distribution in a neighborhood of the mean θ. However, this does “not restrict too much since most of the distributions (such as uniform distribution, normal distribution, exponential distribution, etc.) satisfy this restriction.

**Proposition** **1.** *If ${\left\{S_{i}\right\}}_{i=1}^{n}$ have positive, continuous and bounded probability distribution in a neighborhood of θ, we have the following statements:*

1. *$Y_{n}={arg min}_{S_{i}\in \left\{S_{1},S_{2},...,S_{n}\right\}}\left|S_{i}-\Sigma_{n}\right|$.*
2. *$Y_{n}$ converges almost surely to θ.*
3. *$Y_{n}$ converges in distribution to $N(\theta ,\sigma^{2})$ with the rate of $\sqrt{n}$.*

**Proof.**  We will prove each statement in turn:

1. Define $h\left(x\right)=\sum_{j=1}^{n}{\left(x-S_{j}\right)}^{2}$. We have:
  $$h^{\prime }\left(x\right)=0\Leftrightarrow x=\frac{\sum_{j=1}^{n}S_{j}}{n}=\Sigma_{n}.$$
  So we have:
*x*−∞∑*_n_*+∞*h*′(*x*)−0+*h*(*x*)+∞
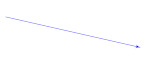

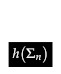

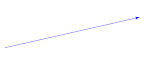
+∞
  From variations of function *h* we imply that the one in $\left\{S_{1},S_{2},...,S_{n}\right\}$ closest to $\Sigma_{n}$ either from the left or the right is the arg min of *h*. On other hand, *h* is symmetric at $\Sigma_{n}$, i.e., for every ε, $h\left(\Sigma_{n}+\varepsilon \right)=h\left(\Sigma_{n}-\varepsilon \right).$ Indeed, define
  $$a_{j}=\frac{1}{n}\left(S_{1}+S_{2}+\cdots +S_{j-1}-\left(n-1\right)S_{j}+S_{j+1}+\cdots +S_{n}\right),$$
  then it is obvious that $\sum_{j=1}^{n}a_{j}=0$. We have
  $$h\left(\Sigma_{n}+\epsilon \right)=\sum_{j=1}^{n}{\left(a_{j}+\epsilon \right)}^{2}=\sum_{j=1}^{n}a_{j}^{2}+n\epsilon^{2}+2\epsilon \sum_{j=1}^{n}a_{j}=\sum_{j=1}^{n}a_{j}^{2}+n\epsilon^{2},$$
  since $\sum_{j=1}^{n}a_{j}=0$.
  Similarly, we have
  $$h\left(\Sigma_{n}-\epsilon \right)=\sum_{j=1}^{n}{\left(a_{j}-\epsilon \right)}^{2}=\sum_{j=1}^{n}a_{j}^{2}+n\epsilon^{2}-2\epsilon \sum_{j=1}^{n}a_{j}=\sum_{j=1}^{n}a_{j}^{2}+n\epsilon^{2}.$$
  So we obtain the first statement.
2. Denote $Z_{n}=Y_{n}-\Sigma_{n}$, we have $Z_{n}$ converges almost surely to 0 and that thus implies $Y_{n}$ converges almost surely to θ. Indeed,
  $$\begin{array}{cc} P(|Z_{n}|\geq \varepsilon ) & =P(|Y_{n}-\Sigma_{n}|\geq \varepsilon )\\ \; & =P\left(\cap_{k=1}^{n}\left\{|S_{k}-\Sigma_{n}\left|\geq \varepsilon \right)\right\}\right)\\ \; & \leq P\left(\cap_{k=1}^{n}\left\{|S_{k}-\theta \left|\geq \varepsilon /2\cup \right|\Sigma_{n}-\theta |\geq \varepsilon /2\right\}\right)\\ \; & \leq P\left(\cap_{k=1}^{n}\left\{|S_{k}-\theta |\geq \epsilon /2\right\}\right)+P(|\Sigma_{n}-\theta \left|\geq \epsilon /2\right)\\ \; & \leq q^{n}+2\sigma \frac{\exp(-n\epsilon^{2}/8\sigma^{2})}{\sqrt{2\pi n}\epsilon }, \end{array}$$
  where $q=P\left\{|S_{k}-\theta |\geq \epsilon /2\right\}<1\forall k=1,...,n$ for all ϵ>0 since by the assumption $S_{k}$ has positive continuous distribution at θ, and $\Sigma_{n}$ converges in distribution to $N(\theta ,\sigma^{2}/n)$ based on central limit theorem and from upper-tail inequality of normal distribution [24] stating that if S∼N(0,1) then for every x>0 we have
  $$P\left(X>x\right)\leq \frac{\exp(-x^{2}/2)}{x\sqrt{2\pi }}.$$
  From theorem 7.5 in [25] we imply $Z_{n}$ converges almost surely to 0 and therefore $Y_{n}$ converges almost surely to θ.
3. The third statement is implied from the fact that $\Sigma_{n}$ converges in distribution to $N(\theta ,\sigma^{2})$ with the rate of $\sqrt{n}$ (central limit theorem) and $Y_{n}$ (the element of $\left\{S_{1},S_{2},...,S_{n}\right\}$ closest to $\Sigma_{n}$) converges in probability to $\Sigma_{n}$ with the rate of *n*. Indeed, to simplify the proof, we assume θ=0, then for any 0<α<1 we have:
  (13) $$\begin{array}{cc} P(n^{\alpha }|Y_{n}-\Sigma_{n}|\geq \varepsilon )= & P(n^{\alpha }|S_{k}-\Sigma_{n}|\geq \varepsilon \forall k=1,2,...n)\\ \; & =P(|\Sigma_{n}|\geq \delta )P(n^{\alpha }|S_{k}-\Sigma_{n}\left|\geq \epsilon \forall k=1,2,..,n\right)\\ \; & +P(|\Sigma_{n}|<\delta )P(n^{\alpha }|S_{k}-\Sigma_{n}\left|\geq \epsilon \forall k=1,2,..,n\right)\\ \; & \leq P(|\Sigma_{n}|\geq \delta )\\ \; & +P(|\Sigma_{n}|<\delta )P(n^{\alpha }|S_{k}-\Sigma_{n}\left|\geq \epsilon \forall k=1,2,..,n\right)\\ \; & =P(|\Sigma_{n}|\geq \delta )\\ \; & +\int_{t=-\delta }^{t=\delta }g_{n}\left(t\right)\prod_{k=1}^{n}\left(1-\int_{s=t-\epsilon n^{-\alpha }}^{s=t+\epsilon n^{-\alpha }}f\left(s\right)ds\right)dt \end{array}$$
  where *f* is density of $S_{k}$ and $g_{n}$ is density of $\Sigma_{n}$.
  With the assumption, $g_{n}$ is positive, continuous and bounded in a neighborhood of 0 as well because it is the density function of $\Sigma_{n}$ which is the average sum of ${\left\{S_{k}\right\}}_{k=1}^{n}$. Let U is the open neighborhood of 0 in which $g_{n}$ and *f* are positive, continuous and bounded. We first fix δ>0, such that [−δ,δ] is in U then if ϵ is small enough we have [−δ−ϵ,δ+ϵ] is in U as well. Therefore we have:
  - $\Delta :=\inf_{\left[-\delta -\epsilon ,\delta +\epsilon \right]}f\left(x\right)>0$;
  - $A:=\sup_{\left[-\delta ,\delta \right]}g_{n}<+\infty$.
  Thus the first component of (13) converges to 0 since $\Sigma_{n}$ converges to 0 a.s; and the second component converges to 0 when ϵ goes to 0 since:
  $$\begin{array}{cc} \; & \int_{t=-\delta }^{t=\delta }g_{n}\left(t\right)\prod_{k=1}^{n}\left(1-\int_{s=t-\epsilon n^{-\alpha }}^{s=t+\epsilon n^{-\alpha }}f\left(s\right)ds\right)dt\\ \; & \leq \int_{t=-\delta }^{t=\delta }g_{n}\left(t\right){\left(1-2\Delta \epsilon n^{-\alpha }\right)}^{n}dt\\ \; & \leq 2\delta A{\left(1-2\Delta \epsilon n^{-\alpha }\right)}^{n}\\ \; & ∼2\delta A{\left(2\Delta \epsilon \right)}^{n^{1-\alpha }}\\ \; & ⟶0\;\mathrm{as}\;n⟶+\infty . \end{array}$$

which ends the proof. □

### 6.2. Two Dimension Case

Now, ${\left\{S_{i}\right\}}_{i=1}^{n}$ are vectors in $R^{2}$. The masks in each nature segmentation problem have a natural “structure” in the meaning that its coordinates are not independent. For the illustrations below, we consider the simplest case where the second component of $S_{i}$ totally depends on its first component, i.e., $S_{i}=\left[x_{i},f\left(x_{i}\right)\right]$ for all *i* where ${\left\{x_{i}\right\}}_{i=1}^{n}$ are i.i.d. mean θ and variance σ and *f* are some functions.

**Figure 12:**
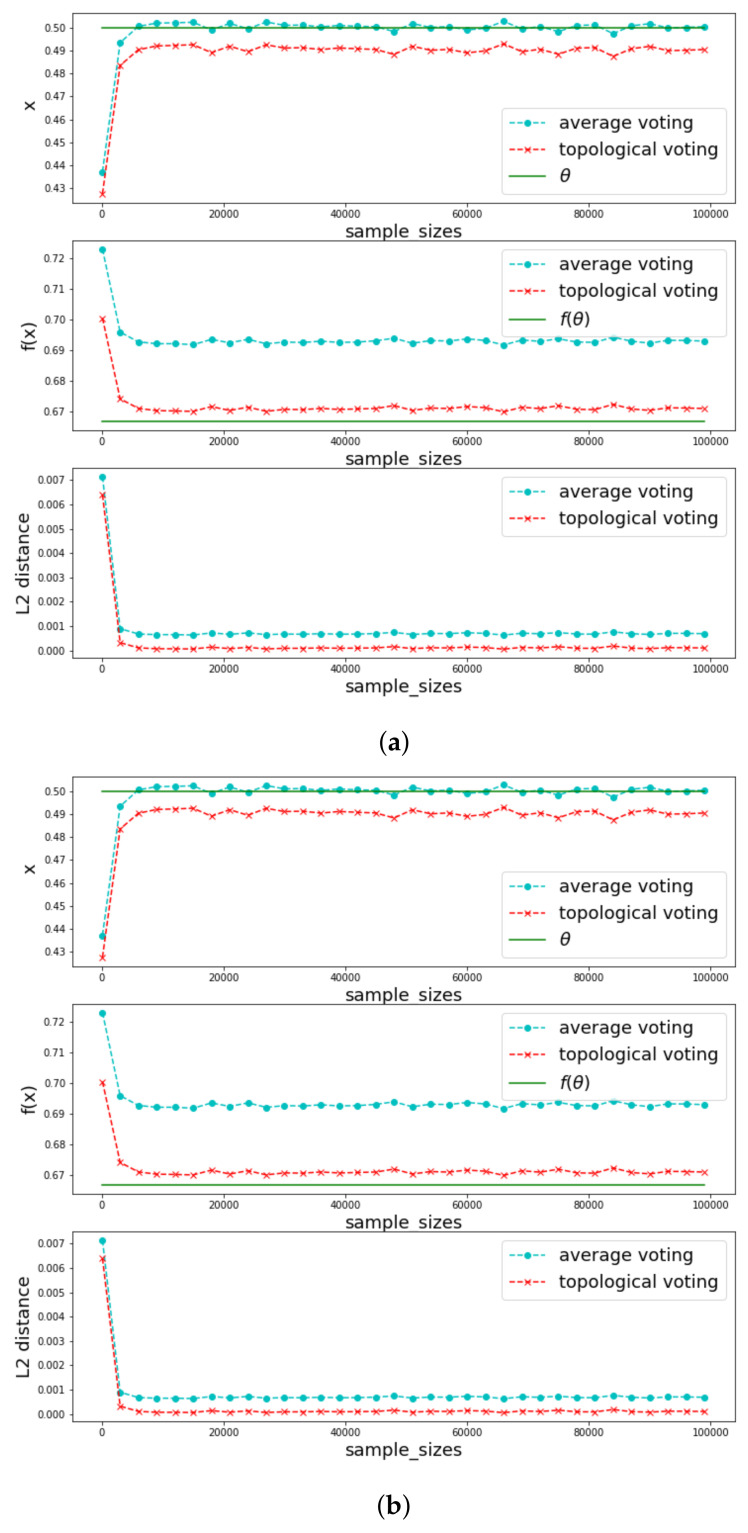
Comparison in two-dimension case with same distribution of $x_{i}$ but different *f*. (**a**) $x_{i}∼U\left(0,1\right)$ for all *i*, f(x)=1/(1+x); (**b**) $x_{i}∼U\left(0,1\right)$ for all *i*, $f\left(x\right)=x^{2}$.

In Figure 12, we vary function *f* to illustrate the different policies of the two voting methods. It can be seen that in the average voting, the first component converges perfectly to the true one but the second component seems to be very far away from the true one, the reason is simple, for most of function *f*, *f* of average is different from average of *f*. Meanwhile, the topological voting takes into account the trade-off between the two components.

**Figure 13:**
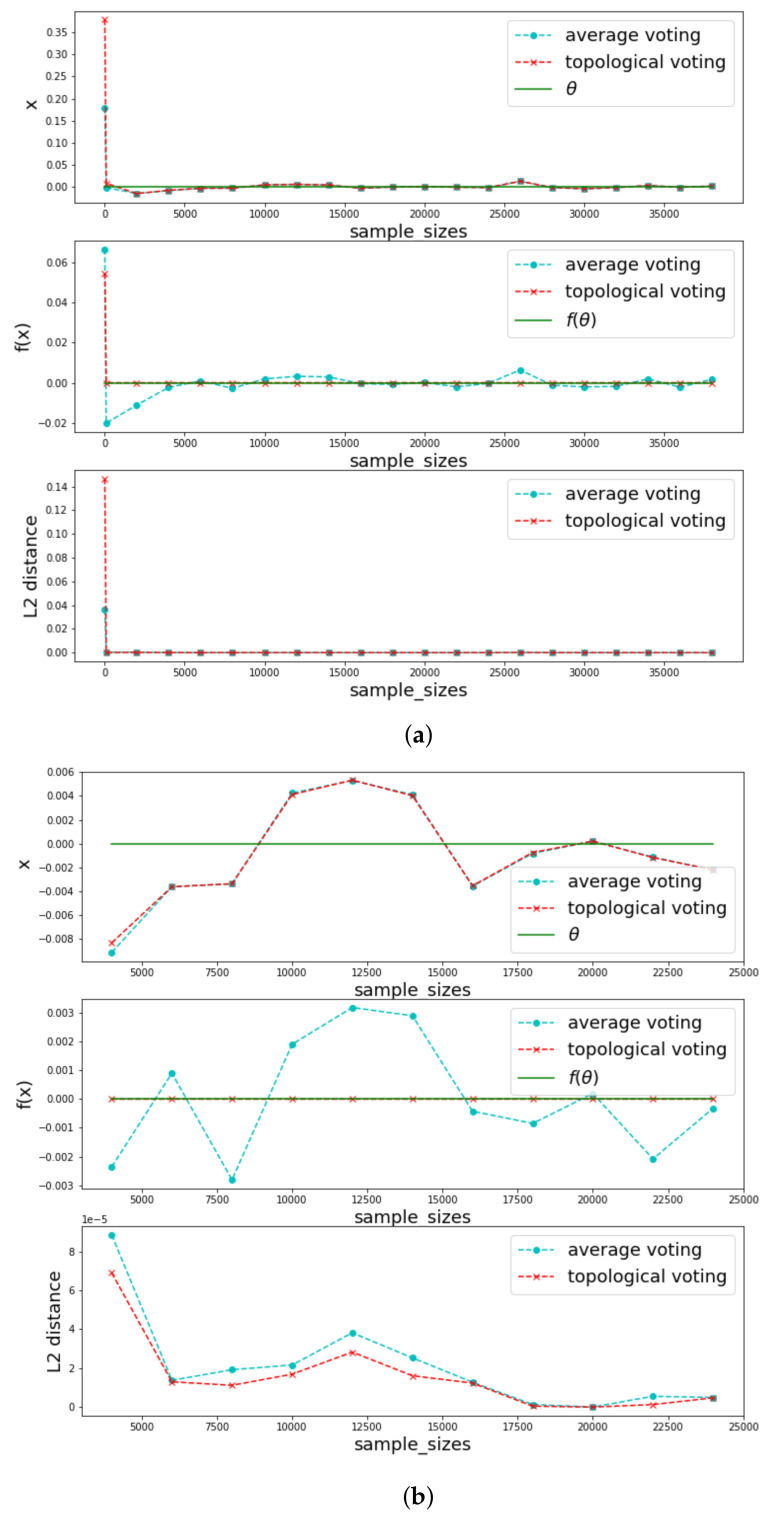
Comparison when *f* is symmetric, $x_{i}∼U\left(-1,1\right),f\left(x\right)=x^{3}$. (**a**) Show for all samples; (**b**) Zoom in a segment of the samples.

In Figure 13, we take a symmetric function *f*. In this case, both of the two components of the result made by topological voting converge very well to the true one because of symmetry property of *f*.

**Figure 14:**
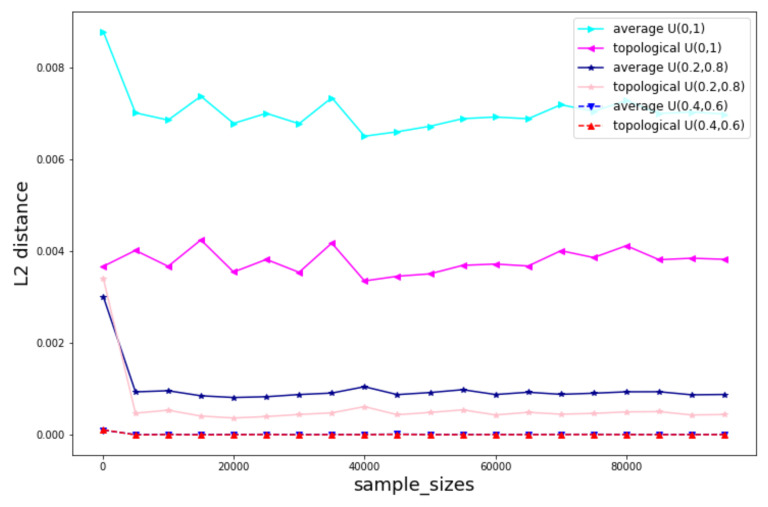
Comparison for different levels of uncertainty of the annotations $x_{i}$. $f\left(x\right)=x^{2}$.

In Figure 14, we keep same *f* but vary distribution of $x_{i}$ to see when the annotations are more or less variance how much different performance are between the two methods. One can see that when we decrease the uncertainty of the annotations, both of the two voting methods behave better especially for the topological voting. The improvement of topological voting to the arithmetical one is significant high when the uncertainty of the annotations is high.

## 7. Conclusions and Future Work

The paper considers various ensemble methods for image segmentation problems. We have presented the three proposed voting methods that take into account the structure of the whole mask. In three concrete examples, many experimental results are shown to prove that our voting methods outperform the classical arithmetical voting methods. We also provided some arguments about the philosophical reason, the mathematical guarantee and the experimental results (for the simplest version) to explain why the proposed topological voting methods behave better than the arithmetical one.

There are several directions in which this work can be taken. One avenue is to extend Proposition 1 for multiple dimension cases and to investigate the theoretical guarantee for the two other versions (the local and hybrid ones). Another avenue is to do more experimental comparisons with more other voting methods, for examples, the nine methods presented in [2].

## Figures and Tables

**Table 1:** Comparison of voting methods on 5 folds for the Salt dataset.

AI Model	Hard Jaccard Score	Interval for *p* (N = 1000, c = 90%)
Fold 1	0.7253	(0.7014, 0.7480)
Fold 2	0.7360	(0.7124, 0.7583)
Fold 3	0.7240	(0.7001, 0.7467)
Fold 4	0.7404	(0.7169, 0.7626)
Fold 5	0.7429	(0.7195, 0.7650)
Soft Arithmetical Voting	0.7813	(0.7590, 0.8020)
Hard Topological Voting	0.7831	( 0.7608, 0.8038)
Soft Topological Voting	0.7847	( 0.7625, 0.8053)

**Table 2:** Comparison of voting methods on 10 folds for the Salt dataset.

AI Model	Hard Jaccard Score	Interval for *p* (N = 1000, c = 90%)
Fold 1	0.7253	(0.7014, 0.7480)
Fold 2	0.7360	(0.7124, 0.7583)
Fold 3	0.7240	(0.7001, 0.7467)
Fold 4	0.7404	(0.7169, 0.7626)
Fold 5	0.7429	(0.7195, 0.7650)
Fold 6	0.7371	(0.7135, 0.7594)
Fold 7	0.7479	(0.7246, 0.7699)
Fold 8	0.7358	(0.7122, 0.7581)
Fold 9	0.7426	(0.7192, 0.7647)
Fold 10	0.7086	(0.6843, 0.7317)
Arithmetical (soft) voting	0.7877	(0.7656, 0.8082)
Topological (soft) voting	0.7996	(0.7779, 0.8197)
Topological (hard) voting	0.8001	(0.7784, 0.8201)
Hybrid voting (threshold = 1.2)	0.8009	(0.7793, 0.8209)
Hybrid voting (threshold = 2)	0.8018	(0.7802, 0.8218)
Hybrid voting (threshold = 3)	0.8027	(0.7811, 0.8226)
Hybrid voting (threshold = 4)	0.7998	(0.7781, 0.8199)
Hybrid voting ($n_{\mathrm{selected}}=4$)	0.8019	(0.7803, 0.8219)
Hybrid voting ($n_{\mathrm{selected}}=5$)	0.8000	(0.7783, 0.8200)
Hybrid voting ($n_{\mathrm{selected}}=7$)	0.7979	(0.7762, 0.8180)

**Table 3:** Comparison of voting methods on 10 folds for the Salt dataset, using binary accuracy score.

AI Model	Binary Accuracy Score	Interval for *p* (N = 1000, c = 90%)
Fold 1	0.9334	(0.9192, 0.9453)
Fold 2	0.9356	(0.9215, 0.9473)
Fold 3	0.9373	(0.9234, 0.9488)
Fold 4	0.9288	(0.9142, 0.9411)
Fold 5	0.9315	(0.9171, 0.9435)
Fold 6	0.9297	(0.9152, 0.9419)
Fold 7	0.9374	(0.9235, 0.9489)
Fold 8	0.9296	(0.9151, 0.9418)
Fold 9	0.9306	(0.9161, 0.9427)
Fold 10	0.9367	(0.9228, 0.9483)
Arithmetical voting	0.9421	(0.9287, 0.9531)
Topological voting		
(using binary accuracy)	0.9412	(0.9277, 0.9523)
Hybrid voting (threshold = 1.5)	0.9417	(0.9282, 0.9527)
Hybrid voting (threshold = 2)	0.9418	(0.9283, 0.9528)
Hybrid voting (threshold = 3)	0.9421	(0.9287, 0.9531)

**Table 4:** Comparison of voting methods on 10 folds for the Face dataset.

	MobileNet Model	EfficientNet Model
Fold 1	0.7572	0.7827
Fold 2	0.7495	0.7845
Fold 3	0.7525	0.7843
Fold 4	0.7527	0.7805
Fold 5	0.7625	0.7849
Fold 6	0.7588	0.7878
Fold 7	0.7611	0.7879
Fold 8	0.7553	0.7917
Fold 9	0.7637	0.7788
Fold 10	0.7604	0.7795
Arithmetical (soft) Voting	0.7909	0.8035
Topological (soft) Voting	0.7832	0.7990
Topological (hard) Voting	0.7833	0.7985
	Score of voting on all 20 segmentors	
		Interval for *p* (N = 1000, c = 90%)
Arithmetical (soft) Voting	0.8088	(0.7875, 0.8285)
Topological (soft) Voting	0.8043	(0.7828, 0.8242)
Topological (hard) Voting	0.8032	(0.7816, 0.8231)
Hybrid voting (threshold = 1.5)	0.8092	(0.7879, 0.8289)
Hybrid voting (threshold = 2)	0.8093	(0.7880, 0.8289)
Hybrid voting (threshold = 3)	0.8092	(0.7879, 0.8289)
Hybrid voting ($n_{\mathrm{select}}=10$)	0.8085	(0.7871,0.8282)
Hybrid voting ($n_{\mathrm{select}}=15$)	0.8090	(0.7877, 0.8287)
Hybrid voting ($n_{\mathrm{select}}=17$)	0.8091	(0.7878, 0.8287)

**Table 5:** Comparison of voting methods on 15 folds for the DRIVE dataset.

AI Model	Hard Jaccard Score	Interval for *p* (N = 2000, c = 90%)
Fold 1	0.6159	(0.5978, 0.6337)
Fold 2	0.6339	(0.6160, 0.6515)
Fold 3	0.6116	(0.5935, 0.6294)
Fold 4	0.6197	(0.6016, 0.6374)
Fold 5	0.6282	(0.6102, 0.6458)
Fold 6	0.6224	(0.6043, 0.6401)
Fold 7	0.6105	(0.5924, 0.6283)
Fold 8	0.6105	(0.5924, 0.6283)
Fold 9	0.6175	(0.5994, 0.6353)
Fold 10	0.6145	(0.5964, 0.6323)
Fold 11	0.6204	(0.6023, 0.6381)
Fold 12	0.6133	(0.5952, 0.6311)
Fold 13	0.6139	(0.5958, 0.6317)
Fold 14	0.6241	(0.6061, 0.6418)
Fold 15	0.6117	(0.5936, 0.6295)
Local topological voting (radius = 20)	0.6347	(0.6168, 0.6523)
Arithmetical (soft) voting	0.6364	(0.6185, 0.6540)
Local topological voting (radius = 10)	0.6384	(0.6205, 0.6560)
Local topological voting (radius = 5)	0.6391	(0.6212, 0.6566)
Hybrid voting (radius = 20, threshold = 2)	0.6399	(0.6220, 0.6574)
Hybrid voting (radius = 5, threshold = 2)	0.6412	(0.6233, 0.6587)
Hybrid voting (radius = 10, threshold = 2)	0.6418	(0.6239, 0.6593)

## Data Availability

Exclude this statement.
